# Suicidal ideation and attempts in population-based samples of women: temporal changes between 1989 and 2015

**DOI:** 10.1186/s12889-019-6685-5

**Published:** 2019-03-29

**Authors:** Solveig Lövestad, Jesper Löve, Marjan Vaez, Margda Waern, Gunnel Hensing, Gunilla Krantz

**Affiliations:** 1 Department of Community Medicine and Public Health, Sahlgrenska Academy at the University of Gothenburg, Box 453, 405 30 Göteborg, Sweden; 2 Department of Clinical Neuroscience, Division of Insurance Medicine, Karolinska Institutet, Berzelius väg 3, 171 77 Stockholm, Sweden; 3 Department of Psychiatry and Neurochemistry, Sahlgrenska Academy at University of Gothenburg, SU Sahlgrenska, 41345 Göteborg, Sweden

**Keywords:** Suicidal ideation, Suicide attempts, Sociodemographic factors, Women, Population-based study, Temporal change

## Abstract

**Background:**

Little is known about temporal changes in the prevalence of self-reported suicidal ideation and attempts within general populations of women. The aim of this study was to assess the prevalence of self-reported suicidal ideation and attempts over a 26 year period (1989–2015) among women from the general population aged 20–49 years. A further aim was to investigate associations between sociodemographic factors and lifetime suicidal ideation over this study period.

**Methods:**

A total of 2072 structured personal interviews were performed with a stratified population-based sample of women between 1989 and 2015. Questions about lifetime suicidal ideation and attempts as well as sociodemographic factors were assessed at four data collection waves. Lifetime prevalence of suicidal ideation and attempts were compared through analysis of differences between two independent proportions and their 95% Confidence Intervals (CI). Associations between sociodemographic factors and lifetime suicidal ideation were estimated by weighted odds ratios (OR).

**Results:**

Women aged 20–30 years reported higher lifetime prevalence of suicidal ideation in 2013–2015 compared to 1989–1991 (45 and 33% respectively). Rates of lifetime suicide attempts remained similar between these time points (3.5 and 3.1% respectively). Women aged 31–49 years reported higher lifetime prevalence of suicidal ideation in 2013–2015 compared to 2000–2002 (35.4 and 23.1% respectively). In this age group, lifetime suicide attempts increased from 0.0% in 2000–2002 to 3.6% in 2013–2015. Women aged 20–30 years who were single, unemployed or had low educational attainment had higher OR of lifetime suicidal ideation compared to the reference categories in most of the study waves. In 2013–2015, young students had lower OR of lifetime suicidal ideation (OR 0.34; 95% CI 0.17–0.69) compared to those with employment. Women aged 31–49 years, who were single, had higher OR of lifetime suicidal ideation (OR 2.61; 95% CI 1.06–6.44) than married, cohabiting women and this was observed in 2013–2015.

**Conclusion:**

The results raise a general concern about an increasing trend in suicidal ideation among young and middle-aged women. The current study expands on previous research by demonstrating that sociodemographic factors may show changing patterns in the associations with lifetime suicidal ideation over time.

## Background

Suicidal *ideation* (or suicidal ‘thoughts’) and *attempts* are known to be strongly associated with completed suicide [1] and contribute to a large burden of disease in terms of adverse psychosocial impacts, long-term disability and increased health care costs [2, 3]. In high income countries, men outnumber women in suicide deaths [3] whereas suicide ideation and attempts are found to be far more common in women [4, 5].

The prevalence of attempted suicide can be studied either by the use of register studies which include all suicide attempts treated in the health-care system, or by population based surveys involving questionnaires or interviews [6]. Most previous research focusing on suicide attempts in Sweden has involved register data [7, 8]. These studies show that between the mid 1990’s and the early 2000s, there was a substantial increase of attempted suicides among women aged 15 to 24 years whereas no increase was observed among women 25 years or older [7, 8]. In their study, Bogdanovica and her colleagues [8] found that among women aged 15–24 years, suicide attempts rates rose from 324.9 per 100,000 in 1989 to 369.4 per 100,000 in 2003. Since 2007, suicide attempt rates have decreased among young women, although they are still higher than in 1987 [9].

While national registers provide extremely valuable data, they lack information about suicide attempts that do not lead to the use of health care services. It has previously been observed that only 50–60% of all suicide attempts are known to the health care systems [6]. Thus, far too little attention has been paid to potential period changes of self-reported suicide attempts among women in general, and in young women outside the health care setting in particular. In addition, no previous study in Sweden has investigated whether the prevalence of self-reported suicidal ideation among young women has increased during the last decades. Studying trends in suicidal ideation is important due to its association with suicide attempts and completed suicide [1], but also in the light of an increasing concern that the mental health of young women is deteriorating [7]. To our knowledge, only one previous population based study in Sweden made a comparison of the prevalence of self-reported suicidal ideation over time [10]. This study presented prevalence figures for men and women as a composite group and showed a decrease in lifetime suicidal ideation between 1986 and 1996 from 33.3 to 21.1% [10]. Although research performed in other high-income countries used repeated measures of suicidal ideation over time, most of these studies have used prevalence figures for men and women as a composite group [11, 12]. These studies did not find any significant change in suicidal ideation between 1990/92 and 2001/03 [12] and 2001/02 and 2011/12 respectively [11]. However, one previous U.S study reported a decrease in lifetime suicidal ideation among women between 1991 and 1992 and 2001–2002 (from 8.2 to 7.1%), while lifetime prevalence of self-reported suicide attempts remained unchanged during this period [13].

The etiology of suicidal ideation is multifactorial and a wide range of sociodemographic factors seem to be of importance, either directly or indirectly by influencing individual’s susceptibility to suicidal ideation [3, 14]. Since the effect of sociodemographic factors on suicidal ideation may be altered through policies and public health interventions [14], it is important to include these factors in research in this field. Earlier population-based studies focusing on women, have demonstrated associations between suicidal ideation and younger age [15], lack of a stable relationship [16, 17], lack of a stable employment [16] and lower educational attainment [16]. Several studies also suggest that university students are a potential risk group [18–20]. However, while these studies have reported on the association between sociodemographic factors and suicidal ideation, only a few studies have investigated the association between sociodemographic factors and suicidal ideation across time among women from the general population [10]. Temporal trends of disease occurrence and risks among different social groups in a population are likely to be dependent on time and place [21]. Thus, historical or social events that occur in a given time period, such as an economic recession may alter the prevalence of suicidal ideation within specific risk groups [14]. This indicates the need to conduct stratified analyses in order to identify temporal changes in the relationship between sociodemographic factors and suicidal ideation in specific risk groups.

The aim of this study was to assess the prevalence of self-reported suicidal ideation and attempts over a 26 year period (1989–2015) in two groups of women from the general population aged 20–30 and 31–49 years. A further aim was to investigate associations between sociodemographic factors and suicidal ideation over this period.

## Methods

### Study design and data collection

This study is part of the four-wave, longitudinal population-based project titled “Women and alcohol in Gothenburg” (WAG) initiated in 1986. The project involved a two-stage stratification procedure with a) an initial postal screening questionnaire (Screening Women and Alcohol in Gothenburg, SWAG), followed by b) structured personal interviews with a stratified sample in the second stage. This two-stage procedure is relevant in epidemiological studies focusing on uncommon conditions such as alcohol-related disorders in women, for which this study was initially designed [22]. The main purpose of this two-stage procedure was to increase the number of individuals with alcohol-related problems, while keeping the numbers of interviews at a reasonable level [22].

The SWAG questionnaire was developed for screening of alcohol-related problems, containing 13 items with a four choice answering mode. This screening questionnaire has been published and described more in detail in a previous article by Spak and Hällström [22]. For the stratification procedure, each question was dichotomized with a negative statement scoring 0 points and a positive statement scoring 1 point, making the maximum total score 13 points. Based on the scores obtained in the SWAG, women were selected for face-to-face interviews by a three category, stratified random sampling. In the first wave, the answers were grouped into three categories: 0 point (no alcohol related problem), 1–3 points (possible alcohol related problems) and ≥ 4 points (probable alcohol related problems) [23]. Out of these groups, all respondents with a SWAG score of ≥4 points, a random quarter of those with 1–3 points and a random one-fifteenth of those who scored 0 points were invited for interview [23]. In the second and third wave, the stratification groups consisted of ≥5, 1–4 and 0 points [24]. The increased cut-off level of ≥5 points was based on an increased alcohol consumption observed among younger women [25]. In the two first waves, a random selection of those who had not responded to the SWAG questionnaire were invited for interview in order to increase the numbers and statistical power. In the fourth and latest wave, due to low response rate in SWAG, all women born in 1993 who returned the SWAG questionnaire were invited to the interview and no stratification procedure was applied.

Those included in the stratified sample were invited by letter to take part in an interview, followed by written reminders and if necessary, telephone calls to non-responders. The same procedure was applied for participants invited to follow-up interviews. Respondents who preferred not to participate in a long face-to-face interview were offered either a shorter version of the interview, a telephone interview or in earlier waves (1989–1991, 1994–1998, 2000–2002), a postal questionnaire. Since questions about suicidal ideation and attempts were not included in the shorter version of the interview, this study includes data from long interviews only.

### Study population

The study population includes women born in 1965, 1970, 1975, 1980 and 1993 who were at the time of the respective examination registered in Central and Western districts in Gothenburg, Sweden’s second largest city. In order to increase the number of participants in the fourth wave of the study, women born in 1993 who were registered residents in Northern and Eastern districts were also invited to participate.

The overall participation rates at each screening and interview wave are presented in Table 1. In 1986, the first SWAG questionnaire was mailed out to all women (*n* = 673) born in 1965, with a response rate of 67.9%. A stratified random sample of 128 respondents were invited for interview and between 1989 and 1991, 74.2% (*n* = 95) of the women completed a long baseline interview. In the second wave in 1994–1998, 2910 screening questionnaires were sent out to all women born in 1970 and 1975 with a response rate of 77.2%. Out of the stratified sample, 543 women participated in their first long interview between 1994 and 1998. In 2000, the screening questionnaire was mailed to 1103 women born in 1980 with a response rate of 75.2%. Out of the stratified sample, 284 long baseline interviews were performed between 2000 and 2002. Finally, in the fourth wave in 2013, the screening questionnaire was sent out to 1687 women born in 1993, out of which 33.9% responded to the questionnaire. After excluding those who refrained further participation (*n* = 4), all 568 women who had responded to the SWAG were invited to take part in the interview and 171 women completed a long baseline interview between 2013 and 2015. All women who took part in a baseline interview were invited to follow-up interviews in subsequent waves. In total 2072 baseline and follow-up interviews were performed between 1989 and 2015. The four waves of data collection are henceforth labelled as W1 (performed during 1989–1991), W2 (1994–1998), W3 (2000–2002) and W4 (2013–2015).

**Table 1:** Screening and interview stages of WAG 1986 to 2015: study population, weighted and unweighted numbers and prevalence (%)

Stage1**:** Screening questionnaire and stratified sample invited to interview					Stage 2**:** Completed long baseline and follow-up interviews: weighted and unweighted numbers (Completed interviews unweighted Total *N* = 2072)							
Unweighted numbers and %					W1. 1989–1991 Baseline		W2. 1994–1998 Baseline and follow-up		W3.2000–2002 Baseline and follow-up		W4.2013–2015 Baseline and follow-up	
					*20–30 years: weighted N = 352*		*20–30 years: weighted N = 2240*		*20–30 years: weighted N = 2089*		*20–30 years: N=171* ^*b*^	
									*31–49 years: weighted N = 216*		*31–49 years: weighted N = 1344*	
Year of birth	Screening year	Mailed screening questionnaire	Response rate	Invited to interview	Unweighted	Weighted	Unweighted	Weighted	Unweighted	Weighted	Unweighted	Weighted
		N	n (%)	n	n (%)	n	n (%)	n	n (%)	n	n (%)	n
1965	1986	673	457 (67.9)	128	95 (74.2)^a^	352	85	349	53	216	31	165
1970, 1975	1995	2910	2247 (77.2)	829	NA		543 (66.0)^a^	1891	415	1604	244	920
1980	2000	1103	829 (75.2)	491	NA		NA		284 (57.8)^a^	485	151	259
1993	2013	1687	572 (33.9)	568	NA		NA		NA		171 (30.1)^a^	NA^b^

*NA* Not Applicable, not selected for interview

^a^Baseline interview

^b^Cohort born in 1993 was not stratified for interview and therefore not weighted. Numbers and % are presented with real numbers and %

### Analysis on differences between long and short interviews

An analysis on the difference between those who completed the long and short interviews showed that women who had completed the long interviews were older, had a higher educational attainment and had a somewhat higher alcohol consumption compared to those who had completed the short interviews.

### Attrition analysis

An analysis performed in a previous study [26] showed no difference in sociodemographic variables (age, marital status, number of children, education and employment status for women and her partner) between those who did and did not respond to SWAG. This type of attrition analysis was possible to perform in the first to waves, when women from the stage 1 attrition group were invited for interview in stage 2, thus answering to a range of background factors during the interview. Telephone interviews with non-responders in previous waves indicated shortage of time as a common reason for declining participation [27].

### The interview

Interviews were conducted after obtaining oral, informed consent in W1-W3 and written informed consent in W4. The interviews were performed by health care professionals and social workers with several years of work experience. A psychiatrist trained the interviewers in the use of the interview questionnaire, as well as classification of psychiatric conditions in accordance with the Diagnostic and Statistical Manual of Mental Disorders (DSM III-R and DSM-IV). A psychiatrist with extensive clinical experience was available for consultation regarding the diagnostic procedures. The interview included questions about sociodemographic factors, childhood conditions, family relations, sexual abuse, intimate partner violence, work-related questions, alcohol consumption, mental health problems and suicide-related behaviors. Structured interviews were conducted either face-to-face at the respondent’s home or at the University of Gothenburg, or by telephone. Interviews lasted for about 1.5 to 3 h.

### Variables

#### Suicidal ideation and attempts

Questions about suicidal ideation and attempts were based on Paykel et al. [28] and Meehan et al. [29] using the following three questions: (1) ‘Have you *ever* had thoughts of taking your life, even if you would not really do it? (suicidal ideation), (2) Have you *ever* reached the point that you seriously considered taking your life, and perhaps made plans how you would go about doing it? (suicidal ideation), (3) Have you *ever* made an attempt to take your life? (suicide attempt). During W2 and W3, the women in the follow-up interviews were asked if they had experienced such thoughts or attempts *during the past 5 years* (instead of *ever*). This time frame was changed in the follow-up interviews in W4 to *‘ever’*, to be consistent with the baseline interviews. All women were asked at both baseline and follow-up if they had experienced questions 1 to 3 above *during the past 12 months*.

In this study, a positive response to either question 1 or 2 during the *past 12 months* was considered as experiencing suicidal ideation during this time period. A positive response to either question 1 or 2 for *ever/ during past 5 years* was considered as experiencing suicidal ideation *earlier in life*. In the next step, changes in prevalence were analyzed using *lifetime* experience of suicidal ideation which was considered when a woman gave a positive response to either of the two merged variables *past 12 months* or *earlier in life*. The same procedure was applied for question 3 (suicide attempts). If values were missing on both *past 12 months* and *earlier in life*, the value was coded as missing. If a negative response (‘no’) on one of the questions and a missing value on another, the answer was coded as ‘no’.

#### Sociodemographic factors

The following sociodemographic factors were included at the four waves of data collection: *education level* (≤ 9 years, 10–12 years (high school) and > 12 years). *Relationship status* was divided into three groups: (1) married, cohabiting, registered partnership (2) widowed, single, never married, non-cohabiting partner and (3) divorced, separated. For the purpose of this paper, all women in category 1 were categorized as ‘married/ cohabiting’ and those in category 2 were categorized as ‘single’. *Current occupation* was tricotomized: (1) those who were working half-time or more (‘employed’), (2) homeworkers, unemployed, women who responded ‘not working because of other reasons’ as well as those on disability pension or sickness absence exceeding 3 months (‘unemployed’), (3) women answering that they were studying half-time or more (‘students’). Participants on parental leave were categorized based on their occupation prior to parental leave.

### Statistical analysis

Analyses were carried out in SPSS 24 using the Complex Samples Plan. This type of analysis adjusts for weights and *“…stratification of the sampling design to produce unbiased national estimates of population means and frequencies from the sample after taking into account weights for over-or undersampling of specific groups”* pp. 232 [30]. Descriptive statistics were presented with unweighted and weighted prevalence (%) and 95% confidence intervals (CI) regarding the sampling fractions according to the scores obtained in SWAG. Since no randomized selection based on the SWAG scores was performed with the cohort born in 1993, no weights for oversampling of alcohol related problems were applied. To test for significant differences in prevalences (Δ) of lifetime suicidal ideation and attempts, 95% CIs were computed in W1 and W4 for women aged 20–30 years, and in W3 and W4 for women aged 31–49 years. [31]. Bivariable associations between each sociodemographic factor and lifetime suicidal ideation were estimated using logistic regression with weighted odds ratios (OR) and 95% CI.

## Results

### Distribution of sociodemographic factors at each data collection wave

The proportion of women with higher education increased in both age groups during the study period, while the proportion who were married or cohabiting decreased (Table 2). Over the 26 year study period, the proportion of women aged 20–30 years who were in paid employment decreased. Among women aged 31–49 years, there was an increase (between W3 and W4) in the proportion of those who had a paid employment.

**Table 2:** Distribution of sociodemographic factors among women 1989 to 2015. Weighted and unweighted numbers and prevalence (%)

Included birth cohorts	W1 1989–1991		W2 1994–1998		W3 2000–2002				W4 2013–2015			
	20–30 years		20–30 years		20–30 years		31–49 years		20–30 years		31–49 years	
	1965		1965/70/75		1970/75/80		1965		1993		1965/70/75/80	
Sociodemographic factors	(Unweighted *N* = 95) Weighted *N* = 352		(Unweighted *N* = 628) Weighted *N* = 2240		(Unweighted *N* = 699) Weighted *N* = 2089		(Unweighted *N* = 53) Weighted *N* = 216		Unweighted *N* = 171^b^		(Unweighted *N* = 426) Weighted *N* = 1344	
	(n) n^a^	%^a^	(n) n^a^	%^a^	(n) n^a^	%^a^	(n) n^a^	%^a^	n^b^	%^b^	(n) n^a^	%^a^
Education level												
> 12 years education	(30) 128	36.4	(286) 1152	51.4	(274) 1052	50.3	(23) 95	44.0	112	65.5	(332) 1053	78.4
10–12 years education (high school)	(56) 197	56.0	(297) 974	43.5	(383) 968	46.3	(26) 105	48.0	56	32.7	(89) 283	21.0
≤ 9 years compulsory school	(7) 22	6.3	(44) 110	4.9	(41) 68	3.3	(4) 16	7.4	3	1.8	(5) 8	0.6
Missing values	(2) 5	1.4	(1) 4	0.2	(1) 1	0.0	(0) 0	0.0	0	0.0	(0) 0	0.0
Current occupation												
Employed	(70) 263	74.7	(302) 1159	51.8	(380) 1315	63.0	(41) 158	73.1	52	30.4	(374) 1173	87.3
Student	(17) 69	19.6	(242) 804	35.9	(264) 595	28.5	(5) 14	6.5	104	60.8	(11) 23	1.7
Unemployed	(5) 11	3.1	(83) 275	12.3	(55) 179	8.5	(7) 44	20.4	15	8.8	(41) 148	11.0
Missing values	(3) 9	2.6	(1) 2	0.1	(0) 0	0.0	(0) 0	0.0	0	0.0	(0) 0	0.0
Relationship status												
Married/ cohabiting	(62) 229	65.1	(270) 1139	50.9	(310) 1163	55.7	(41) 180	83.3	34	19.9	(334) 1046	77.9
Divorced/ separated	(10) 45	12.8	(74) 289	12.9	(91) 271	13.0	(8) 23	10.6	7	4.1	(47) 187	13.9
Single	(23) 78	22.2	(284) 812	36.3	(297) 654	31.3	(4) 13	6.0	130	76.0	(44) 110	8.2
Missing values	(0) 0	0.0	(0) 0	0.0	(1) 1	0.0	(0) 0	0.0	0	0.0	(1) 1	0.1

(N) (n) Unweighted total N and numbers

^a^Weighted numbers and prevalence (%) based on total weighted N

^b^Cohort born in 1993 was not stratified for interview and therefore not weighted. Numbers and % are presented with real numbers and %

### Prevalence of lifetime suicidal ideation and attempts

More women aged 20–30 years reported lifetime prevalence of suicidal ideation in W4 compared to W1 (45% compared with 33%; Δ 12.1%; 95% CI 3.2–20.9) (Table 3). For lifetime suicide attempts, rates were similar in W4 and W1 (3.5% compared with 3.1%; Δ 0.4%; 95% CI -2.7 - 4.6). Among those aged 31–49 years, a higher proportion reported lifetime prevalence of suicidal ideation in W4 than in W3 (35.4% compared with 23.1%; Δ 12.3%; 95% CI 5.7–18.0). In W4, 3.6% (95% CI 1.7–7.5) of the women in this age group reported lifetime suicide attempts.

**Table 3:** Lifetime suicidal ideation and attempts among women 1989 to 2015. Weighted numbers, prevalence (%)^a^ and differences between proportions (Δ)^b^

Suicidal ideation and attempts	W1 1989–1991		W2 1994–1998		W3 2000–2002		W4 2013–2015		Difference between two independent proportions
	20–30 years, weighted *N* = 352		20–30 years, weighted *N* = 2240		20–30 years, weighted *N* = 2089 \31–49 years, weighted *N* = 216		20–30 years *N* = 171^d^ 31–49 years, weighted *N* = 1344		
	(n) n^c^	% (95% CI)^c^	(n) n^c^	% (95% CI)^c^	(n) n^c^	% (95% CI)^c^	(n) n^c^	% (95% CI)^c^	Δ % (95% CI)
*Aged 20–30 years*									
Suicidal ideation									
Lifetime	(34) 116	33.0 (21.0–47.6)	(280) 906	40.4 (35.3–45.5)	(226) 484	23.2 (19.7–26.9)	77^d^	45.0 (37.7–52.6)^d^	Δ _W4-W1_ 12.1 (3.2–20.9)
No	(57) 226	64.2 (49.7–76.5)	(345) 1319	58.9 (53.5–64.1)	(470) 1602	76.7 (72.9–80.1)	94^d^	55.0 (47.4–62.3)^d^	
Missing values	(4) 10	2.8	(3) 15	0.7	(3) 3	0.1	0	0.0	
Suicide attempts									
Lifetime	(5) 11	3.1 (1.1–8.8)	(39) 93	4.2 (2.8–6.0)	(24) 51	2.4 (1.5–3.9)	6^d^	3.5 (1.6–7.6)^d^	Δ _W4-W1_ 0.4 (− 2.7–4.6)
No	(86) 331	94.0 (87.5–97.3)	(160) 563	25.1 (20.6–30.3)	(669) 2032	97.3 (95.8–98.3)	131^d^	76.6 (69.7–82.4)^d^	
Missing values	(4) 10	2.8	(429) 1584	70.7	(6) 6	0.3	34^d^	19.9^d^	
*Aged 31–49 years*									
Suicidal ideation									
Lifetime					(10) 50	23.1 (10.1–44.6)	(162) 476	35.4 (29.1–42.2)	Δ _W4-W3_ 12.3 (5.7–18.0)
No					(42) 162	75.0 (54.0–88.4)	(263) 866	64.5 (57.6–70.7)	
Missing values					(1) 4	1.9	(1) 2	0.1	
Suicide attempts									
Lifetime					(0) 0	0.0	(16) 49	3.6 (1.7–7.5)	NA
No					(52) 212	98.1 (87.7–99.7)	(195) 479	35.7 (30.0–41.7)	
Missing values					(1) 4	1.9	(215) 816	60.7	

(n) Unweighted numbers based on unweighted total N

*NA* Not Applicable

^a, b^Prevalence and differences between two independent proportions (Δ) are presented with 95% Confidence Intervals (CI)

^c^Weighted numbers and prevalence (%) based on total weighted N

^d^Cohort born in 1993 was not stratified for interview and therefore not weighted. Numbers and % are real numbers and %

### Associations between sociodemographic factors and lifetime suicidal ideation

In W2 and W3, women aged 20–30 years with 9 years of compulsory school or less had higher OR of lifetime suicidal ideation compared to those with more than 12 years of education (OR 3.86; 95% CI 1.68–8.89 and OR 8.00; 95% CI 3.40–18.78 respectively) (Table 4). In W4, women with high school education (10–12 years) had a higher OR of lifetime suicidal ideation (OR 3.37; 95% CI 1.72–6.59) compared to those with more than 12 years of education. Likewise, being a student and being single both showed increased OR for lifetime suicidal ideation in W2 and W3 compared to the reference categories. Being a student was associated with lower OR of lifetime suicidal ideation in W4 (OR 0.34; 95% CI 0.17–0.69), as compared to being employed. The OR of suicidal ideation at any point in life was more than 4-fold among unemployed women aged 20–30 years in W2. Women aged 31–49 years who were single had more than two times higher OR of lifetime suicidal ideation (OR 2.61; 95% CI 1.06–6.44) compared to women who were married or cohabiting (Table 5).

**Table 4:** Associations between sociodemographic factors and lifetime suicidal ideation, women aged 20–30 years 1989 to 2015

Sociodemographic factors	W1 1989–1991			W2 1994–1998			W3 2000–2002			W4 2013–2015		
	(Total weighted sample *N* = 352)			(Total weighted sample *N* = 2240)			(Total weighted sample *N* = 2089)			(Total sample *N* = 171)^a^		
	Suicidal ideation: no/yes			Suicidal ideation: no/yes			Suicidal ideation: no/yes			Suicidal ideation: no/yes		
	Weighted numbers and OR 95% CI			Weighted numbers and OR 95% CI			Weighted numbers and OR 95% CI			Unweighted ^a^ numbers and OR 95% CI		
	No	Yes *n* = 116	OR (95% CI)^b^	No	Yes *n* = 906	OR (95% CI)^b^	No	Yes *n* = 484	OR (95% CI)^b^	No	Yes *n*=77^a^	OR (95% CI)^b^
Education level												
> 12 years education	75	53	1	672	474	1	911	141	1	73	39	1
10–12 years (high school)	133	55	0.58 (0.15–2.25)	618	347	0.79 (0.50–1.25)	660	306	3.01 (1.89–4.79)	20	36	3.37 (1.72–6.59)
≤ 9 years compulsory school	14	8	0.81 (0.10–6.22)	29	81	3.86 (1.68–8.89)	30	37	8.00 (3.40–18.78)	1	2	3.74 (0.33–42.60)
Missing values	4	0		0	4		1	0		0	0	
Current occupation												
Employed	170	84	1	787	368	1	1079	233	1	21	31	1
Student	46	23	1.01 (0.26–3.98)	442	357	1.73 (1.11–2.81)	393	202	2.38 (1.51–3.75)	69	35	0.34 (0.17–0.69)
Unemployed	6	5	1.69 (0.17–16.94)	91	179	4.23 (2.23–8.01)	130	49	1.73 (0.79–3.77)	4	11	1.86 (0.52–6.73)
Missing values	4	4		0	2		0	0		0	0	
Relationship status												
Married/ cohabiting	157	67	1	751	384	1	976	186	1	20	14	1
Divorced/ separated	31	9	0.68 (0.11–4.37)	148	136	1.80 (0.87–3.70)	225	45	1.04 (0.51–2.12)	0	7	–
Single	38	40	2.47 (0.53–11.50)	420	386	1.80 (1.12–2.88)	401	253	3.31 (2.02–5.42)	76	56	1.08 (0.50–2.34)
Missing values	0	0		0	0		0	0		0	0	

^a^Cohort born in 1993 was not stratified for interview and therefore not weighted. Numbers and % are real numbers and %

^b^Odds Ratio (OR) with 95% Confidence Intervals (CI)

**Table 5:** Associations between sociodemographic factors and lifetime suicidal ideation, women aged 31–49 years 2000 to 2015

Sociodemographic factors	W3 2000–2002			W4 2013–2015		
	(Total weighted sample *N* = 216)			(Total weighted sample *N* = 1344)		
	Suicidal ideation: no/yes			Suicidal ideation: no/yes		
	Weighted numbers and OR 95% CI			Weighted numbers and OR 95% CI		
	No	Yes *n* = 50	OR (95% CI) ^a^	No	Yes *n* = 476	OR (95% CI)^a^
Education level						
> 12 years education	70	25	1	674	377	1
10–12 years (high school)	80	21	0.73 (0.07–7.76)	190	93	0.88 (0.44–1.77)
≤ 9 years compulsory school	12	4	0.93 (0.05–16.25)	2	6	5.38 (0.73–39.48)
Missing values	0	0		0	0	
Current occupation						
Employed	124	30	1	783	388	1
Student	13	1	0.32 (0.02–4.41)	9	14	3.14 (0.72–13.77)
Unemployed	25	19	3.14 (0.22–45. 17)	74	74	2.00 (0.76–5.29)
Missing values	0	0		0	0	
Relationship status						
Married/ cohabiting	127	49	1	696	348	1
Divorced/ separated	22	1	–	122	66	1.08 (0.49–2.37)
Single	13	0	–	48	62	2.61 (1.06–6.44)
Missing values	0	0		1	0	

^a^Odds Ratio (OR) with 95% Confidence Intervals (CI)

## Discussion

### Summary of main findings

This is one of the first studies in Sweden assessing period changes of self-reported suicidal ideation and attempts among women from a population-based sample. Our findings show an increase in the prevalence of lifetime suicidal ideation among women aged 20–30 and 31–49 years, as well as an increase of lifetime suicide attempts among women aged 31–49 years. Low educational attainment, being single, lacking employment and being a student, were associated with lifetime suicidal ideation in most of the study waves.

### Prevalence of lifetime suicidal ideation and attempts

We found an increase of lifetime suicidal ideation among both age groups at the end of the study period. Previous population-based studies performed in high-income countries show large disparities in prevalence when it comes to long term trends of suicidal ideation and attempts. Earlier studies have used shorter time periods [10–13] and/ or presented data for men and women as a composite group [10, 11] which hampers direct comparison with our findings. Further, variations between instruments, methodology and time periods under study, as well as cultural differences in participants’ willingness to disclose suicidal ideation make it difficult to determine whether differences in prevalence reflect real variations or are differences due to methodological disparities [3, 32]. Having this said, the increasing prevalence of suicidal ideation found in this study is in accordance with data published by the National Board of Health and Welfare in 2017 [33], showing increasing trends of depression and anxiety among young women in Sweden. Besides individual suffering and pain, raising trends of mental illness, including suicidal ideation, may lead to increased barriers for educational attainment and access to future labour market. Thus, future studies should closely monitor whether the increasing patterns of suicidal ideation persist and try to focus on mechanisms behind this trend. Cordinated and comprehensive interventions using the available knowledge of risk factors for suicidal ideation are needed [3]. This includes early access to help and early detection within the primary health care settings.

### Associations between sociodemographic factors and lifetime suicidal ideation

#### Educational attainment

In the last three waves we found that among young women, low educational attainment was associated with higher OR of lifetime suicidal ideation compared to women with more than 12 years education. This is consistent with previous research [12, 19, 34]. In a study set in Spain, Gabilondo et al. [19] found that men and women with low or middle level education were 4.3 times more likely to report suicidal ideation compared to those with high educational attainment. Higher educational attainment is believed to benefit mental health through the attainment of important advantages which mitigate life stressors, i.e. more economic and social resources as well as better access to and use of mental health services [35]. On the other hand, low educational attainment may lead to increased risk for suicidal ideation through social disadvantage [4]. For instance, the qualification requirements for paid employment have increased, and those without a university degree have less favorable employment conditions and prospects compared to those who do have a university degree [36]. This may affect the mental well-being of young adults [36] and could be one of many explanations for our finding that young women with compulsory school and/or high school education were more likely to report suicidal ideation compared to women with higher educational attainment. Our findings indicate the importance of maintaining generous safety net programs in order to mitigate social disadvantage among those with low educational attainment and subsequently counteract increased suicidal ideation within this group. 

#### Current occupation

Students in the 20–30 years age group had higher OR of lifetime suicidal ideation in 1994–1998 and 2000–2002 than their peers with employment. Earlier research has identified students as a risk group [18, 19, 34]. It is suggested that lack of self-confidence, feelings of worthlessness [20] and having a frail social support network contribute to suicidal ideation among students [37]. Surprisingly, we observed a negative association between student status and suicidal ideation in 2013–2015. It is not clear how to interpret this finding and we have not been able to find previous research in line with this result. However, as social and environmental conditions alter over time and space, it is likely that the relationship between covariates and health outcomes also fluctuates over time [38]. The composition of the group reporting student status has most likely changed since the beginning of the 1990s. For instance, we did not ask the women to specify whether they were studying at the university or whether they were in vocational training, a factor that may differ over time depending on political decisions and changes in the labour market. Since the beginning of the 1990s, the number of students in Sweden has almost doubled and today more than half of the female population aged 25–34 years has a tertiary education [39]. Thus, in the last wave, a large proportion of the young women responding that they were students may already have a high educational attainment and opted for a continuation of their university studies. As those with high educational attainment are less likely to experience suicidal ideation [16], a plausible explanation may be that young students in the last wave were mentally better-off than students in previous cohorts and data collection waves, because they already had a higher educational degree. Further, due to housing shortage, particularly in larger Swedish cities [40], young students might not have the option of moving away from home. Remaining in the parental home may have some benefits such as maintenance of available social networks and support from parents, siblings and friends, which in turn helps to mitigate stressful life events [4].

Unemployed women aged 20–30 years had a higher OR of lifetime suicidal ideation between 1994 and 1998 compared to those with employment. It is likely that this association between unemployment and lifetime suicidal ideation is, at least in part, related to the situation for young adults in the general population at that time. Between 1990 and 1993, the economic recession contributed to a drop in employment rates among all age groups. This was particularly pronounced among people aged 20–24 years [36, 41] with a decline from about 60 to 39% [36]. Additionally, the labour market for young people did not recover to the same extent as it did for other age groups and this may have contributed to an increase in mental health problems [36]. Previous studies have shown associations between unemployment and suicidal ideation among women [16, 42]. However, the influence of unemployment on suicidal ideation may vary according to gender, age and the sociopolitical context including access to social benefits and assistance during fiscal austerity [14]. Strong social safety nets that prevent economic exclusion due to unemployment, together with maintenance (instead of cost-cutting measures) of mental health care services are shown to mitigate the negative mental health effects of unemployment in times of economic recession [43].

#### Relationship status

We found that single women in both age groups had higher OR of lifetime suicidal ideation than those who were married/ cohabiting. However, this association was not constant over the study period. Earlier research has found that women reporting suicidal ideation were less likely to be married or cohabiting [17] and more likely to be divorced or separated [16, 17]. Previous findings show that the association between relationship status and a health outcome is complex and partly explained by socio-economic advantages, in terms of stronger social integration and greater economic resources, that a cohabiting or married relationship provides [44]. The implications of relationship status on health may also depend on the cultural and historical context, as well as the particular cohort under study [44]. For instance, despite changing values, marriage and long-lasting intimate relationships are still regarded as a norm, particularly when it comes to older age groups [45]. Singles have in earlier studies been described (by married, cohabiting people and singles themselves) as more self-centered, less socially mature and envious compared to married, cohabiting people [44, 45] and 40-year-old singles are more harshly judged than 25-year-old singles [44, 45]. As public views may lead to negative self-perceptions and internalized expectations (one ‘has to be cohabiting/ married at a certain age’) [45], this could also be one plausible explanation to why single women aged 31–49 years in our study had higher OR of suicidal ideation than their cohabiting, married counterparts.

### Methodological considerations

The main strength of this study is the fact that we used repeated cross-sectional surveys with the same measures and comparable age groups of women from the general population. Another strength is the use of the same sociodemographic variables when investigating the association between sociodemographic factors and suicidal ideation over a 26 year period. A further strength is that data on suicidal ideation and attempts are based on face-to-face interviews employing standardized questions on suicidal ideation and behavior.

However, some limitations should be borne in mind when interpreting the results. First, the assessment of the occurrence and timing of suicidal ideation and attempts was based on retrospective self-reported information and suicide attempts were not confirmed by medical records or registers. This may have introduced potential problems with under or over reporting and biased recall. For example, previous research suggests that past suicidal ideation is likely to be forgotten, deliberately denied or unconsciously repressed because of earlier painful memories [46]. Second, self-reported information about suicidal ideation and attempts may have been influenced by social desirability bias i.e. the participant’s willingness to report confidential information, which most likely occurs in response to socially sensitive questions [47]. Third, due to low power we explored neither temporal changes in the lifetime prevalence of suicide attempts, nor associations between sociodemographic factors and suicide attempts. Fourth, to be able to analyze trends over time, we kept the sociodemographic variables with exactly the same categories over the four data collection waves. This however, led to small sample sizes (n = < 10) and large confidence intervals for some of the variables. The results therefore need to be interpreted with caution.

Fifth, the response rate in the screening questionnaire as well as the participation rate in the interviews were considerably lower in 2013–2015 compared to earlier waves (33.9 and 30.1% respectively). This may impact on the generalizability of the results in this study. For instance, people with high educational attainment and high socioeconomic status are more likely to participate in scientific studies [48]. As women with low socioeconomic status and educational attainment are more likely to report suicidal ideation than their counterparts [19], the prevalence figures for suicidal ideation and attempts are likely to be underestimated in our study. This is particularly true for the last wave in which a greater proportion had higher education. However, it is also suggested that there is little evidence demonstrating that low response rate leads to substantial bias [48]. Rising numbers of requests to participate in epidemiological studies, together with increasingly complex and demanding research protocols have been suggested as underlying causes for decreasing participation rates [48]. In addition, growing numbers of cell phone users and unlisted telephone numbers make it harder to reach potential participants [48].

## Conclusions

Data from this study raise a general concern about an increasing trend in suicidal ideation among young and middle-aged women. Based on this study, no conclusions can be drawn about the mechanisms behind the changes in association between sociodemographic factors and suicidal ideation over time. However, the results supports clinical and public health focus on younger, socioeconomically disadvantaged women. Further population-based studies with large samples and more frequent measurement occasions are warranted to establish whether changes in suicidal ideation and attempts reflect changes in environmental conditions and life circumstances for women in the general population.

## Abbreviations

CI: Confidence Interval
DSM-III-R: Statistical Manual of Mental Disorders, 3rd Edition Revised
DSM-IV: Statistical Manual of Mental Disorders, Fourth Edition
OR: Odds Ratio
SWAG: Screening Women and Alcohol in Gothenburg
U.S: United States
WAG: Women and Alcohol in Gothenburg

## References

[1] Klonsky ED, May AM, Saffer BY (2016). Suicide, Suicide Attempts, and Suicidal Ideation. Annu Rev Clin Psychol.

[2] Kerkhof A (2012). Calculating the burden of disease of suicide, attempted suicide, and suicide ideation by estimating disability weights. Crisis.

[3] World Health Organization (2014). Preventing suicide - A global imperative.

[4] Nock MK, Borges G, Bromet EJ, Cha CB, Kessler RC, Lee S (2008). Suicide and suicidal behavior. Epidemiol Rev.

[5] Boyd A, Van de Velde S, Vilagut G, de Graaf R, O'Neill S, Florescu S, Alonso J, Kovess-Masfety V (2015). Gender differences in mental disorders and suicidality in Europe: results from a large cross-sectional population-based study. J Affect Disord.

[6] Kjoller M, Helweg-Larsen M (2000). Suicidal ideation and suicide attempts among adult Danes. Scand J Public Health.

[7] Kosidou K, Magnusson C, Mittendorfer-Rutz E, Hallqvist J, Hellner Gumpert C, Idrizbegovic S, Dal H, Dalman C (2010). Recent time trends in levels of self-reported anxiety, mental health service use and suicidal behaviour in Stockholm. Acta Psychiatr Scand.

[8] Bogdanovica I, Jiang G-X, Löhr C, Schmidtke A, Mittendorfer-Rutz E (2011). Changes in rates, methods and characteristics of suicide attempters over a 15-year period: comparison between Stockholm, Sweden, and Würzburg, Germany. Soc Psychiatry Psychiatr Epidemiol.

[9] Guo-Xin Jiang**,** Gergö Hadlaczky**,** Danuta Wasserman. Självmordsförsök i Sverige. Data: 1987-2014.http://ki.se/sites/default/files/sjalvmordsforsok_i_sverige_1987-2014.pdf. Accessed 2 Jan 2019**.**

[10] Renberg ES (2001). Self-reported life-weariness, death-wishes, suicidal ideation, suicidal plans and suicide attempts in general population surveys in the north of Sweden 1986 and 1996. Soc Psychiatry Psychiatr Epidemiol.

[11] Miret M, Caballero FF, Huerta-Ramírez R, Moneta MV, Olaya B, Chatterji S, Haro JM, Ayuso-Mateos JL (2014). Factors associated with suicidal ideation and attempts in Spain for different age groups. Prevalence before and after the onset of the economic crisis. J Affect Disord.

[12] Kessler RC, Berglund P, Borges G, Nock M, Wang PS (2005). Trends in suicide ideation, plans, gestures, and attempts in the United States, 1990-1992 to 2001-2003. J Am Med Assoc.

[13] Baca-Garcia E, Perez-Rodriguez MM, Keyes KM, Oquendo MA, Hasin DS, Grant BF, Blanco C (2010). Suicidal ideation and suicide attempts in the United States: 1991-1992 and 2001-2002. Mol Psychiatry.

[14] Conejero I, Lopez-Castroman J, Giner L, Baca-Garcia E (2016). Sociodemographic Antecedent Validators of Suicidal Behavior: A Review of Recent Literature. Curr Psychiatry Rep.

[15] Kovess-Masfety V, Boyd A, Haro JM, Bruffaerts R, Villagut G, Lepine JP, Gasquet I, Alonso J (2011). High and low suicidality in Europe: a fine-grained comparison of France and Spain within the ESEMeD surveys. J Affect Disord.

[16] Fanous AH, Prescott CA, Kendler KS (2004). The prediction of thoughts of death or self-harm in a population-based sample of female twins. Psychol Med.

[17] Forkmann T, Brähler E, Gauggel S, Glaesmer H (2012). Prevalence of suicidal ideation and related risk factors in the German general population. J Nerv Ment Dis.

[18] Pereira A, Cardoso F (2015). Suicidal ideation in university students: prevalence and association with school and gender. Paideia.

[19] Gabilondo A, Alonso J, Pinto-Meza A, Vilagut G, Fernández A, Serrano-Blanco A, Almansa J, Codony M, Haro JM (2007). Prevalence and risk factors for suicide ideation, plans and attempts in the Spanish general population. Results from the ESEMeD study. Med Clin.

[20] Eskin M, Sun JM, Abuidhail J, Yoshimasu K, Kujan O, Janghorbani M, Flood C, Carta MG, Tran US, Mechri A (2016). Suicidal behavior and psychological distress in university students: a 12-nation study. Arch Suicide Res.

[21] Kuh D, Shlomo Y, Kuh D, Shlomo Y, Susser E (2004). Life course approaches to socioeconomic differentials in health. A Life Course Approach to Chronic Disease Epidemiology.

[22] Spak F, Hallstrom T (1996). Screening for alcohol dependence and abuse in women: description, validation, and psychometric properties of a new screening instrument, SWAG, in a population study. Alcohol Clin Exp Res.

[23] Spak L, Spak F, Allebeck P (1998). Sexual abuse and alcoholism in a female population. Addiction.

[24] Sundin M, Spak F, Spak L, Sundh V, Waern M (2011). Substance use/abuse and suicidal behavior in young adult women: a population-based study. Subst Use Misuse.

[25] Andersson C, Eklund M, Sundh V, Thundal KL, Spak F (2012). Women's patterns of everyday occupations and alcohol consumption. Scand J Occup Ther.

[26] Spak F, Hallstrom T (1995). Prevalence of female alcohol dependence and abuse in Sweden. Addiction.

[27] Hensing G, Spak F, Thundal KL, Ostlund A (2003). Decreased risk of alcohol dependence and/or misuse in women with high self-assertiveness and leadership abilities. Alcohol Alcohol (Oxford, Oxfordshire).

[28] Paykel ES, Myers JK, Lindenthal JJ, Tanner J (1974). Suicidal feelings in the general population: a prevalence study. Br J Psychiatry.

[29] Meehan PJ, Lamb JA, Saltzman LE, O'Carroll PW (1992). Attempted suicide among young adults: progress toward a meaningful estimate of prevalence. Am J Psychiatry.

[30] Saylor J, Friedmann E, Lee HJ (2012). Navigating complex sample analysis using national survey data. Nurs Res.

[31] Newcombe RG (1998). Interval estimation for the difference between independent proportions: comparison of eleven methods. Stat Med.

[32] De Leo D, Cerin E, Spathonis K, Burgis S (2005). Lifetime risk of suicide ideation and attempts in an Australian community: prevalence, suicidal process, and help-seeking behaviour. J Affect Disord.

[33] The National Board of Health and Wefare: Utvecklingen av psykisk ohälsa bland barn och unga vuxna Till och med 2016**.** https://www.socialstyrelsen.se/Lists/Artikelkatalog/Attachments/20785/2017-12-29.pdf. Accessed 2 Jan 2019.

[34] Nock MK, Borges G, Bromet EJ, Alonso J, Angermeyer M, Beautrais A, Bruffaerts R, Wai TC, De Girolamo G, Gluzman S (2008). Cross-national prevalence and risk factors for suicidal ideation, plans and attempts. Br J Psychiatry.

[35] Phillips JA, Hempstead K (2017). Differences in U.S. suicide rates by educational attainment, 2000-2014. Am J Prev Med.

[36] Lager A, Berlin M, Heimerson I, Danielsson M (2012). Young people's health: Health in Sweden: The National Public Health Report 2012. Chapter 3. Scand J Public Health.

[37] Gonçalves A, Sequeira C, Duarte J, Freitas P (2014). Suicide ideation in higher education students: influence of social support. Aten Primaria.

[38] Jan Van den B, Jonathan RB (2013). Epidemiology: principles and practical guidelines.

[39] Statistics Sweden (SCB): Educational attainment of the population; 2016. https://www.scb.se/contentassets/66fa9c1d1f904b4aa22ac4a816d9e9a5/uf0506_2016a01_sm_uf37sm1701.pdf. Accessed 26 Mar 2019.

[40] The Swedish Union of Tenants (Hyresgästföreningen): Unga vuxnas boende. Hur påverkar situationen på bostadsmarknaden unga vuxnas möjligheter att skapa sin egen framtid? https://www.hyresgastforeningen.se/globalassets/globalt-innehall/rapporter/unga-vuxna-2017/unga-vuxnas-boende-2017.pdf. Accessed 8 Oct 2018.

[41] The Public Health Agency of Sweden: Folkhälsan i Sverige Årsrapport; 2014. https://www.folkhalsomyndigheten.se/contentassets/30d592b0c7834256bd893e1ba0d56b6c/folkhalsan-i-sverige-arsrapport-2014.pdf. Accessed 26 Mar 2019.

[42] Legleye S, Beck F, Peretti-Watel P, Chau N, Firdion JM (2010). Suicidal ideation among young French adults: association with occupation, family, sexual activity, personal background and drug use. J Affect Disord.

[43] Van Hal G (2015). The true cost of the economic crisis on psychological well-being: a review. Psychol Res Behav Manag.

[44] Depaulo BM, Morris WL (2005). Target Article: singles in society and in science. Psychol Inq.

[45] Schütz A, Hertel J, Depaulo B, Morris W, Stucke T (2007). She’s single, so what? How are singles perceived compared with people who are married?. Zeitschrift für Familienforschung.

[46] Goldney RD, Winefield AH, Winefield HR, Saebel J (2009). The benefit of forgetting suicidal ideation. Suicide Life Threat Behav.

[47] Althubaiti A (2016). Information bias in health research: definition, pitfalls, and adjustment methods. J Multidiscip Healthc.

[48] Galea S, Tracy M (2007). Participation rates in epidemiologic studies. Ann Epidemiol.

